# Cognitive functioning and brain MRI findings six months after acute COVID-19. A prospective observational study

**DOI:** 10.1016/j.ynirp.2025.100254

**Published:** 2025-03-22

**Authors:** Janne Pihlajamaa, Henriikka Ollila, Juha Martola, Linda Kuusela, Riikka Pihlaja, Annamari Tuulio-Henriksson, Sanna Koskinen, Viljami Salmela, Laura Hokkanen, Marjaana Tiainen, Johanna Hästbacka

**Affiliations:** a HUS Medical Imaging Centre, Radiology, Helsinki University Hospital and University of Helsinki, Helsinki, Finland; b Department of Perioperative and Intensive Care, Helsinki University Hospital and University of Helsinki, Helsinki, Finland; c Department of Psychology and Logopedics, Faculty of Medicine, University of Helsinki, Helsinki, Finland; d Department of Neurology, Helsinki University Hospital and University of Helsinki, Helsinki, Finland; e Anesthesia and Intensive Care, Tampere University Hospital, Wellbeing District of Pirkanmaa and Tampere University, Faculty of Medicine and Health Technology, Tampere, Finland; f Division of Neuropsychology, HUS Neurocenter, Helsinki University Hospital and University of Helsinki, Helsinki, Finland

**Keywords:** COVID-19, MRI, Cognitive functioning, Long-term, Cerebral microbleeds

## Abstract

**Introduction:**

COVID-19 has been linked to many neurological complications, including cognitive impairment and findings in brain imaging. However, limited data exist regarding the link between magnetic resonance imaging (MRI) findings and cognitive functioning in COVID-19 patients.

In this observational prospective study, we investigated the association between brain MRI findings, particularly cerebral microbleeds (CMBs) and white matter hyperintensities (WMHs), and cognitive functioning in COVID-19 survivors.

**Methods:**

Six months after acute COVID-19 diagnosed in 2020, 67 ICU-treated, 44 ward-treated, and 44 home-isolated patients, as well as 48 non-COVID-19 controls, underwent MRI and comprehensive neuropsychological evaluation. We applied multivariable linear regression models to investigate the independent associations of total cognitive score and domain scores separately with CMBs, WMHs and other factors.

**Results:**

Age (p < 0.001, β = −0.36) and educational level (p < 0.001, β = 0.42) predominantly explained the differences in cognitive functioning. A lower total cognitive score was associated with the number of CMBs (p = 0.0016), but not with COVID-19 (p = 0.714). Among COVID-19 patients, treatment in a regular ward (p = 0.007, β = −0.46), a high burden of WMHs (p = 0.004, β = −1.35), and having one to three CMBs (p = 0.01, β = −0.43) were associated with lower total cognitive scores.

**Conclusion:**

We observed a significant association between the presence of CMBs and lower cognitive scores, regardless of COVID-19 history. However, our results do not support CMBs to be independently associated with cognitive functioning. Additionally, WMH burden was associated with lower cognitive scores.

## 1 Introduction

Coronavirus disease 2019 (COVID-19) may lead to acute and prolonged complications including various abnormal brain imaging findings and cognitive dysfunction (Newcombe et al., 2021; Ceban et al., 2022). Several neuroinflammatory mechanisms may contribute to cognitive impairment in COVID-19 (Monje and Iwasaki, 2022), (Saikarthik et al., 2022). The most common imaging findings in the acute phase include infarctions, hemorrhage, white matter hyperintensities (WMHs) and perfusion disorders detected by arterial spin labeling magnetic resonance imaging (MRI). Contrast-enhanced imaging has also revealed leptomeningeal enhancement (Newcombe et al., 2021; Ladopoulos et al., 2021; Gulko et al., 2020; Kremer et al., 2020). Data on radiological findings regarding long-term follow-up are scarce, but studies have reported white matter changes, atrophy in different areas of the brain, and cerebral microbleeds (CMBs) (Hellgren et al., 2021; Lu et al., 2020; Douaud et al., 2022; Vasilev et al., 2023).

CMBs are small traces of hemorrhage visible as signal attenuations in susceptibility-weighted (SWI) MRI sequences (Shams et al., 2015; Lee et al., 2018). The exact underlying pathophysiology of CMBs is still unclear, but CMBs have been reported in many conditions such as hypertension, amyloid angiopathy, Alzheimer's disease, severe acute respiratory distress syndrome, and high-altitude cerebral edema, and, in a small proportion of the population, as incidental findings (Shams et al., 2015; Lee et al., 2018; Fanou et al., 2017; Kallenberg et al., 2008; Riech et al., 2015). The main risk factors for CMBs include high blood pressure, hyperlipidemia, diabetes, male sex, and older age (Shams et al., 2015; Lee et al., 2018). CMBs are associated with cognitive decline in aging individuals and hypertensive patients (Akoudad et al., 2016; Hilal et al., 2014). During or after severe COVID-19, CMBs have been detected in the corpus callosum and particularly in the splenium as well as in the juxtacortical white matter and brainstem, with similar topography as previously described in critical illness and hypoxemia (Newcombe et al., 2021; Kremer et al., 2020; Fanou et al., 2017; Dixon et al., 2020; Ollila et al., 2023; van Lith et al., 2024; Wang et al., 2024).

WMHs appear as signal hyperintensities on T2 weighted brain MRI, typically located in deep white matter. They are a surrogate of small vessel cerebrovascular disease, sharing risk factors with CMBs (Debette and Markus, 2010). The presence of WMHs is associated with an increased risk of both ischemic and hemorrhagic stroke, dementia, and mortality (Debette and Markus, 2010). COVID-19 accelerates the accumulation of white matter changes and general brain atrophy (Douaud et al., 2022; van Lith et al., 2024; Agarwal et al., 2021; Paterson et al., 2020; Radmanesh et al., 2020; Diez-Cirarda et al., 2023), although in a few cases, the number of WMHs has decreased (Radmanesh et al., 2020), suggesting an unclear pathophysiological process.

In COVID-19 patients, few studies have reported associations between MRI findings and cognitive functioning (Hellgren et al., 2021; Douaud et al., 2022; Raman et al., 2021; Cecchetti et al., 2022; Boparai et al., 2023). A large study found an association between the reduction of volume crus II of the cerebellum and greater cognitive decline, while other studies reported mixed or no associations (Douaud et al., 2022; Boparai et al., 2023).

Our group has previously shown that ICU-treated and hospitalized but non-ICU-treated COVID-19 patients presented with more cognitive impairment compared to home-isolated patients (Ollila et al., 2022); in terms of MRI findings, we found that the ICU-treated patients had more CMBs in deep areas, particularly in the splenium of the corpus callosum, compared to other COVID-patients and non-COVID-19 controls. However, the overall number or prevalence of CMBs between different severity groups did not differ, although a higher number of days with supplementary oxygen was independently associated with CMBs (Ollila et al., 2022, 2023).

Given the previous findings of long-term cognitive impairment in COVID-19 survivors (Ollila et al., 2022) and findings in MRI, we now aimed to further assess whether cognitive impairment is associated with the presence and location of CMBs, or the extent of WMHs, and the role of COVID-19 as an explanatory factor. We hypothesized that while the number or location of CMBs might be associated with cognitive impairments, they would not be independent predictors when factors such as age and comorbidities are taken into account. We expected to find an association of WMHs with cognitive impairment also in this population, but we hypothesized that COVID-19 would not have an independent role in cognitive impairment.

## 2 Methods

This is a pre-planned sub-study of the RECOVID study project (ClinicalTrials.gov NCT04864938), a multidisciplinary observational cohort study that examines long-term neurological, cognitive, psychological, cardiac, and pulmonary outcomes of ICU-treated, ward-treated, and home-isolated COVID-19 patients and non-COVID-19 controls. We report the results according to the Strengthening the Reporting of Observational Studies (STROBE) guideline (Cuschieri, 2019).

### 2.1 Participants and study procedures

A detailed description of the formation of the study population has been published (Ollila et al., 2022, 2023). Briefly, we included patients with Finnish as their primary language, who had tested positive for SARS-CoV-2 in a polymerase chain reaction test or an antibody test and were aged 18 or older. Exclusion criteria were pregnancy, previous significant neurological diseases such as severe traumatic brain injury (TBI) or stroke, progressive memory disorder, developmental intellectual disability, impairment in hearing or vision, contraindications for MRI, such as severe claustrophobia, cardiac pacemaker, or MRI-incompatible foreign material in the body. This sub-study involves subjects who underwent both brain MRI and comprehensive neuropsychological testing.

Eligible patients diagnosed with acute COVID-19 in the Helsinki University Hospital district between March 12th and December 31st, 2020, were recruited to the study. We used the level of care (intensive care unit [ICU]; regular hospital ward [WARD], or home [HOME]) as a surrogate for the severity of the acute disease.

The ICU group was recruited by a written invitation to consecutive eligible ICU survivors after hospital discharge. The WARD group was recruited at hospital wards during the acute disease or at a pulmonary outpatient follow-up appointment. We recruited the non-hospitalized HOME group and a group of non-COVID-19 controls (CONTROL) via media announcements.

Brain imaging and neuropsychological evaluation occurred six months after hospital discharge (ICU and WARD groups) or six months after a positive COVID-19 test (HOME group).

The ethics committee of Helsinki University Hospital approved the study protocol (HUS-1949-2020). All subjects gave written informed consent for participation in the study and were treated according to the principles of the Declaration of Helsinki.

### 2.2 Clinical data

Clinical data on hospitalized patients were collected from the electronic patient records (Uranus™, CGI, Montreal, Canada; Apotti, Epic™, Verona, USA) and intensive care patient data management systems (Apotti, Epic™, Verona, USA; PICIS™, Wakefield, USA; Clinisoft, Clinisoft Oy, Kuopio, Finland). We collected information about hospital treatment parameters such as duration of treatment and background information (weight, height and comorbidities) and ICU-related parameters.

For the HOME and CONTROL groups, we recorded comorbidities from patient records and during the interviews. By the time of the study, no antibody test was available to exclude a previous SARS-CoV-2 infection. However, we did not expect undetected infections in the CONTROL group, as isolation was common and PCR testing was actively used for any symptomatic individuals.

### 2.3 MRI data

Details of the imaging protocol have been published before (Ollila et al., 2023). An experienced neuroradiologist (J.M.), with over 15 years of experience in neuroradiology, interpreted the imaging results blinded to the clinical details or severity group of the participants. Infarctions were detected in only 2.6 % of participants (Ollila et al., 2023), and we chose not to focus on them in the current analysis.

To identify the locations of CMBs, we used the Microbleed Anatomical Rating Scale (Gregoire et al., 2009). During the analysis, we detected an abnormal number of CMBs in the splenium of the corpus callosum region, prompting us to include this location in our recording. We assessed the WMHs in T2 and FLAIR sequences and categorized them using the Fazekas scale (Fazekas et al., 1987) ranging from 0 to 3 (0 = no changes, 1 = mild, 2 = moderate, and 3 = severe changes).

The 3D FLAIR was imaged with a TR 4800 ms, TE 320 ms, TI 1650ms, FOV 240 × 240 mm, in-plane resolution 1 × 1.2 mm, slice thickness 1.2 mm, TSE 140, SENSE factor 2 (phase direction) and 1.5 in slice direction and total scan time 4 min 48 s. The axial SWI was imaged with a TR 31 ms, TE 7.2, 13.4, 19.6, 25.8 ms, FOV 240 × 194 mm, in-plane resolution 0.6 × 0.6 mm, slice thickness 2 mm, compressed sense factor 4 and total scan time 3 min 31 s. The T2 3D was imaged with a TR 2500 ms, TE 331 ms, FOV 230 × 230 mm, in-plane resolution 0.8 × 0.85 mm, slice thickness 0.7 mm, TSE 117, SENSE factor 2 (phase direction) and 1.5 in slice direction and total scan time 5 min 7 s. Other sequence information can be found in the supplementary material (S12).

### 2.4 Cognitive data

We evaluated cognitive functioning using a 2-h face-to-face comprehensive neuropsychological evaluation. From the complete neuropsychological data, scores for three cognitive domains: attention, memory, and executive functioning, were calculated. The formation of these domains was based on the standardized Z-scores of the respective neuropsychological raw test scores (see our previous publication for more details on neuropsychological methods (Ollila et al., 2022)). We also calculated a total cognitive score as the sum of the domain scores. In all scores, a higher value indicates better performance. The total cognitive score was used as the main outcome variable. We also analyzed the association between MRI findings and the three cognitive domains. The neuropsychological evaluation methods are further detailed in the supplementary material (S11).

### 2.5 Statistical analysis

Our primary objective was to examine the associations between cognitive functioning and brain MRI findings. To identify factors related to cognitive functioning, we grouped patients according to quartiles of their total cognitive score. We selected variables for further analysis using univariable comparisons between different quartiles.

In our analysis, we treated CMBs as a categorical variable stratified into three categories based on the number of CMBs (0, 1–3, and 4 or more). We used this approach because the CMB distribution in our data was highly skewed, with a large proportion of participants having no CMBs and a small proportion having a high number of CMBs.

We present continuous variables as means and standard deviations (SD) or medians and interquartile ranges (IQR) and categorical variables as numbers and percentages. In univariable comparisons, we used the Chi-squared test or Fisher's exact test for categorical variables and the non-parametric Kruskal-Wallis test for continuous variables with non-normal distributions.

To study the association of imaging findings with the total cognitive score, we used a linear regression model to adjust for potential confounding factors. We selected factors with a significance level of p < 0.2 between Z-score quartiles in univariable comparisons to the model together with previously known risk factors (Newcombe et al., 2021; Debette and Markus, 2010; Gorelick et al., 2011; Luchsinger and Mayeux, 2004; O'Brien et al., 2003; Rahman and Sathi, 2021). The variables were investigated in hierarchical linear regression: model 1 including the demographic variables only, model 2 adding comorbidities, model 3 adding the history of COVID-19, and model 4 adding the MRI variables. Additionally, we included CMB locations in the model, to evaluate their potential influence on the total cognitive score and its subdomains. We set the level of statistical significance at p < 0.05. Given that multiple tests were conducted, we applied False Discovery Rate (FDR) correction to control for false positives. The FDR-corrected threshold for significance was set at < 0.05. We present standardized beta estimates as an indicator of effect size, representing the average change in the outcome variable associated with a one-unit change in the predictor variable, holding all other variables constant. In regression models, we excluded two patients with unusually high numbers of CMBs (N = 140 and N = 361) and one patient with the lowest total cognitive score (−23.35) as clear outliers. Additionally, one patient was excluded from the analysis of CMBs and WMHs due to findings suggestive of diffuse axonal injury (DAI). We used R studio version 4.1.1 (Oct 8, 2021) and Jamovi project (2021) (Version 2.2) [Computer Software, retrieved from https://www.jamovi.org] in the analyses.

## 3 Results

**Fig. 1:**
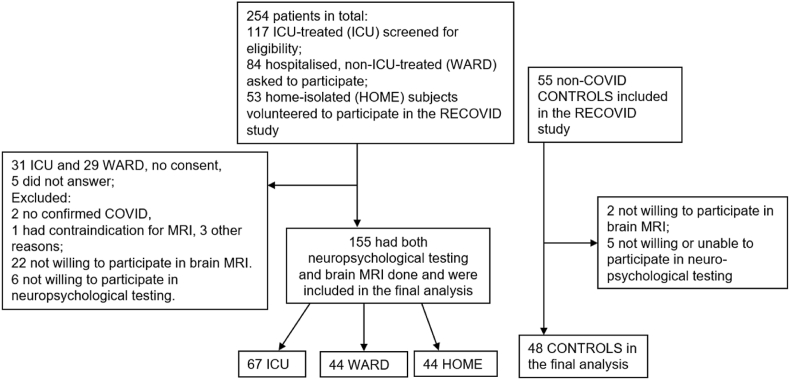
Flow chart showing the number of study subjects included in the final analysis.

The study population consisted of 67 ICU-treated, 44 ward-treated, 44 home-isolated patients, and 48 non-COVID-19 controls (Fig. 1). In Finland, vaccinations against SARS-CoV-2 started at the end of December 2020. Consequently, all participants were unvaccinated. The Wuhan and Delta variants were the dominant variants at the time of the study (Gupta et al., 2023). Typing of the variant was unavailable.

### 3.1 Study outcomes

Subjects in the poorest performing quartile in cognitive assessment were older (p = 0.002), more often men (p = 0.003), and had a higher prevalence of hypertension (p < 0.001), hypercholesterolemia (p < 0.001), diabetes (p < 0.001), and had the least years of education (p < 0.0001) than the other quartiles (Table 1). The level of care was significantly different between quartiles (p = 0.002), with the highest proportion of ICU-treated subjects in the lowest quartile (Table 1).

**Table 1:** Patient characteristics, level of care, imaging findings and cognitive functioning organized according to quartiles of the total cognitive score. The quartiles range from the lowest (1) to the highest (4) performing quartile.

Total cognitive score quartile	1, n = 51	2, n = 51	3, n = 51	4, n = 50	χ^2^/F	p
**Sex male, n (%)**	34 (67)	28 (55)	18 (35)	18 (36)	14.3	**0.003**
**Age, years, median (IQR)**	63 (53–73)	58 (48–68)	51 (39–53)	46 (35–57)	24.2	**< 0.001**

**Level of care:**					37.7	**0.002**
**ICU, n (%)**	28 (55)	16 (31)	16 (31)	7 (14)		
**WARD, n (%)**	11 (22)	13 (25)	11 (22)	9 (18)		
**HOME, n (%)**	1 (2)	7 (14)	14 (27)	22 (44)		
**CONTROL, n (%)**	11 (22)	15 (29)	10 (20)	12 (24)		

**Education years, median (IQR)**	12 (9–15)	15 (13–20)	15 (13–17)	16 (14–18)	17.0	**< 0.001**
**Hypertension, n (%)**	31 (61)	21 (41)	10 (20)	7 (14)	31.1	**< 0.001**
**Hypercholesterolemia, n (%)**	19 (37)	15 (29)	3 (6)	6 (12)	19.6	**< 0.001**
**Diabetes, n (%)**	13 (25)	8 (16)	1 (2)	2 (4)	17.6	**< 0.001**

**CMB number**					2.14	0.039^†^
**0 CMB, n (%)**	30 (59)	37 (73)	38 (75)	42 (82)	8.45	0.038^†^
**1**–**3 CMBs, n (%)**	16 (31)	10 (20)	10 (20)	7 (14)	4.82	0.103
**≥4 CMBs, n (%)**	5 (10)	4 (8)	2 (4)	1 (2)	3.42	0.330
**CMB location**						
**Deep, n (%)**	7 (14)	6 (12)	3 (6)	4 (8)	2.09	0.589
**Splenial, n (%)**	4 (8)	2 (4)	2 (4)	1 (2)	2.14	0.546
**Lobar, n (%)**	17 (33)	11 (22)	9 (18)	7 (14)	6.19	0.104
**Infratentorial, n (%)**	5 (10)	3 (6)	5 (10)	2 (4)	1.93	0.589

**Fazekas scale, n (%)**					37.1	**< 0.001**
**0, n (%)**	3 (6)	4 (8)	10 (20)	16 (32)		
**1, n (%)**	37 (73)	47 (92)	38 (75)	32 (64)		
**2, n (%)**	8 (16)	0 (0)	2 (4)	2 (4)		
**3, n (%)**	3 (6)	0 (0)	0 (0)	0 (0)		
**Infarctions, n (%):**	2 (4)	2 (4)	0 (0)	0 (0)	4.04	0.257
**Total cognitive score, median (IQR)**	−7.2 (−12.8 to −1.6)	−0.9 (−2.6–0.8)	3.2 (1.8–4.6)	6.3 (4.3–8.3)		
**Memory, median (IQR)**	−2.2 (−4.5 to −0.1)	−0.5 (−2.6–2.4)	0.9 (−1.0–2.8)	2.5 (0.9–4.1)		
**Attention, median (IQR)**	−2.8 (−5.3 to −0.3)	−0.3 (−1.7–1.1)	1.0 (−0.1–2.1)	2.0 (1.2–2.8)		
**Executive functioning, median (IQR)**	−2.5 (−6.2–1.3)	0.0 (−1.8–1.8)	1.2 (0.0–2.4)	2.0 (1.2–2.8)		

IQR interquartile range, ICU intensive care unit, CMB cerebral microbleed. Bolding indicates statistically significant p-values.

^†^ non-significant after FDR correction.

One patient in this group had DAI and was therefore excluded from this category.

**Fig. 2:**
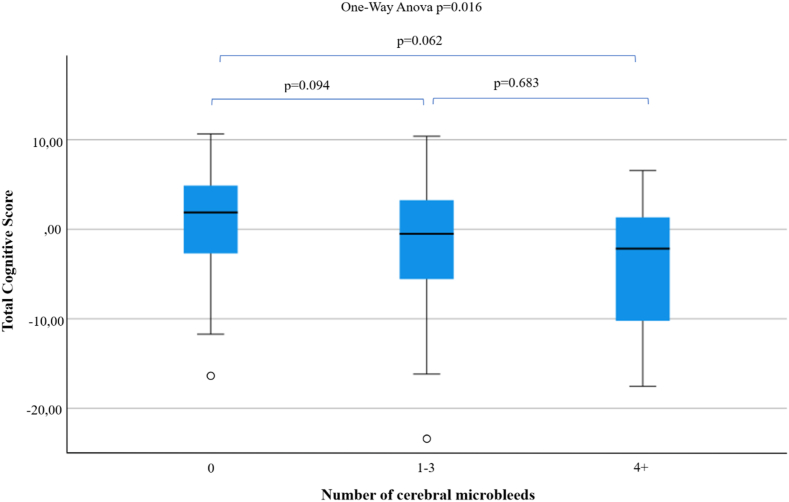
Total cognitive score according to the number of CMBs in the whole study population (n = 203). Whiskers indicate the minimum and maximum observed values within 1.5 times the interquartile range (IQR). The ANOVA test revealed a statistically significant overall effect; however, pairwise comparisons showed no significant differences. IQR interquartile range.

A higher proportion of subjects in the poorest performing quartile had CMBs (p < 0.038, p (FDR) = 0.066) and a higher Fazekas scale (p < 0.001). CMB category (0, 1–3 or ≥4 CMBs) was significantly associated with total cognitive score (p = 0.016) (Fig. 2). However, in pairwise comparisons, no differences emerged between CMB categories.

### 3.2 Linear regression models

Table 2 presents the linear regression models for factor tested in association with the total cognitive score. Additional models for the total cognitive score and the subdomain scores can be accessed in the supplementary material.

**Table 2:** Linear regression models for factor tested in association with the total cognitive score.

A: The whole study population, except the 3 outliers that were excluded, n = 200, R^2^ = 0.515				
Factors tested for association with cognition	β	SE	t	p
Age:	−0.3552	0.0618	−5.779	**< 0.001**
Education:	0.4110	0.0541	7.649	**< 0.001**
Male sex:	−0.0957	0.1074	−0.896	0.371
Hypertension:	−0.1850	0.1394	−1.335	0.183
Hypercholesterolemia:	−0.0543	0.1581	−0.346	0.730
Diabetes:	−0.1258	0.2041	−0.620	0.536
History of COVID-19:	−0.0479	0.1255	−0.385	0.701
Fazekas (ref = 0):				
1	−0.1316	0.1477	−0.896	0.371
2	−0.2620	0.2785	−0.947	0.345
3	−1.3465	0.4670	−2.903	**0.004**
CMB number (ref = 0):				
1-3	−0.1858	0.1316	−1.422	0.157
≥4	0.0780	0.2413	−0.326	0.745

B: COVID-19 patients only n = 152, R^2^ = 0.534				
Factors tested for association with cognition, COVID-19 patients only	β	SE	t	p
Age:	−0.2937	0.0768	−3.855	**< 0.001**
Education:	0.4548	0.0647	7.082	**< 0.001**
Male sex:	0.0418	0.1285	0.328	0.743
Level of care (ref = home):				
Ward	−0.4641	0.1694	−2.763	**0.007**
ICU	−0.2957	0.1722	−1.731	0.086
Hypertension:	−0.1299	0.1597	−0.820	0.413
Hypercholesterolemia:	−0.0547	0.1777	−0.310	0.757
Diabetes:	−0.1442	0,2156	−0.675	0.501
Fazekas (ref = 0):				
1	−0.0608	0,1755	−0.350	0.727
2	−0.3836	0,3037	−1.274	0.205
3	−1.5780	0,5613	−2.837	**0.005**
CMB number (ref = 0):				
1-3	−0.3727	0,1522	−2.472	**0.015**
≥4	0.0760	0,2449	0.313	0.755

C: ICU patients only n = 65, R^2^ = 0.477				
Factors tested for association with cognition, ICU patients only	β	SE	t	p
Age:	−0.29945	0.1092	−2.8063	**0.007**
Education:	0.38813	0.1090	3.6431	**< 0.001**
Male sex:	−0.00457	0.2416	−0.0194	0.985
Hypertension:	−0.14213	0.2448	−0.5943	0.555
Hypercholesterolemia:	0.17924	0.2914	0.6294	0.532
Diabetes:	−0.71746	0.3489	−2.1042	0.040^†^
Deep CMB:	−0.13072	0.3992	−0.3351	0.739
Splenial CMB:	1.03274	0.5283	2.0005	0.051
Lobar CMB:	−0.61488	0.2813	−2.2368	0.030^†^
Infratentorial CMB:	0.24464	0.5953	0.4205	0.676

β standardized β estimate, SE standardized standard error, ref reference level, CMB cerebral microbleed, ICU intensive care unit. Bolding indicates statistically significant p-values.

^†^ non-significant after FDR correction.

In the linear regression model for the whole study population, except the 3 outliers that were excluded (Table 2A), age (p < 0.001, β = −0.36) and years of education (p < 0.001, β = 0.41) were the most significant explanatory factors for the total cognitive score. Fazekas scale 3 of WMHs was found to be significantly associated with the total cognitive score (p = 0.004, β = −1.27) but only three patients were in that category. Age, sex, and education accounted for 47 % of the variance in the total cognitive score (Table S1, model 1). Incorporating COVID-19 into the model did not lead to a significant increase in the explained variance (Table S1, model 3) and the history of COVID-19 was not statistically significantly associated with the total cognitive score (p = 0.701). Hypertension, diabetes, and hypercholesterolemia collectively accounted for an additional 2 % of the variance (Table S1, model 3), while MRI variables (CMB amount and Fazekas) accounted for an additional 3 %, but the effects were statistically non-significant (Table S1, model 4). Comorbidities or sex were not independently associated with the total cognitive score.

Among COVID-19 patients (Table 2B), the level of care had a significant association with the total cognitive score. Being in the WARD group compared to the HOME group was significantly associated with lower cognitive scores (p = 0.007, β = −0.46); being in the ICU group was not independently associated (p = 0.086, β = −0.30).

We found an association between the presence of CMBs and lower total cognitive scores. The effect was significant in the 1–3 CMBs category only (p = 0.015, β = −0.37). Having 4 or more CMBs (n = 11) was not independently associated with lower total cognitive score. No significant associations between the locations of CMBs and the total cognitive score or the sub-domains were found (Tables S2–S8).

We conducted a focused subgroup analysis on the locations of CMBs in patients admitted to the ICU (Table 2C) and found an association between the presence of lobar CMBs (p = 0.03, p (FDR) = 0.0725, β = −0.61) and the total cognitive score. In domain-specific analyses, the association of lobar CMBs existed in executive functions (Table S7). CMBs located in the deep or infratentorial regions were not significantly associated with the total cognitive score or the subdomains (Table 2C and S2-S8). Diabetes was associated with a lower total cognitive score (p = 0.04), but this association did not remain statistically significant after applying FDR correction. ICU-related parameters were not significantly associated with total cognitive score (Table S9).

## 4 Discussion

In this observational prospective single-center study we investigated the associations between cognitive functioning and brain MRI findings among subjects who had recovered from the acute phase of COVID-19 as well as non-COVID-19 controls. Our main finding was that the number of CMBs was associated with a lower total cognitive score in this population of patients six months after the acute phase of COVID-19 and controls when adjusting for confounders such as age, sex, educational level and comorbidities. The history of COVID-19 was, however, not a statistically significant independent factor in explaining the total cognitive score. Among COVID-19 patients, those with 1–3 CMBs exhibited a lower total cognitive score. The location of CMBs was not significantly associated with the total cognitive score. Additionally, the burden of WMHs, classified according to the Fazekas scale, was associated with worse cognitive functioning.

Our findings regarding the association between a higher number of CMBs and lower cognition in the total study population are consistent with prior studies. Research conducted before the COVID-19 pandemic has shown that the presence of multiple CMBs is associated with a decline in the total cognitive score [18], domain-specific scores including executive functions, attention, information processing, memory, visuo-construction and motor speed (Hilal et al., 2014; Poels et al., 2012; Werring et al., 2004), as well as lower Mini-Mental State Examination scores (Akoudad et al., 2016). Similarly, WMHs are among the strongest radiological predictors of cognitive functioning in patients without COVID-19 ^23,42,43^. This was found also in our study.

In patients with a history of COVID-19, we assessed the associations of the subdomains of attention, memory and executive functioning with CMB locations. We found a weak association between lobar CMBs and executive functions, but the effect remained statistically significant after the FDR correction only in the ICU-treated patients. The number of subjects with CMBs in each location was small which may explain the lack of clear findings. Few studies have focused on the connection between focal MRI abnormalities such as CMBs and cognitive functioning in COVID-19, and the results have been conflicting (Boparai et al., 2023). A recent longitudinal imaging study of 785 UK biobank participants revealed that the SARS-CoV-2 positive group showed a significantly greater cognitive decline, associated with greater atrophy of crus II of the cerebellum (Douaud et al., 2022). A study of 86 post-COVID syndrome patients and 36 healthy controls found significant relationships with gray matter atrophy and cognitive dysfunction, mostly with attention, working memory and processing speed (Diez-Cirarda et al., 2023). A study of 36 COVID-19 patients found that a higher volume of white matter hyperintensities in the left parieto-occipital region was associated with poorer immediate and delayed memory 10 months after hospital discharge. No other significant correlations were observed (Cecchetti et al., 2022). In a study of 58 COVID-19 patients, SWI sequences revealed increased bilateral thalamic T2∗ signal and slightly increased volume of white matter hyperintensities. However, these MRI findings showed no correlation with cognitive functioning measured by Montreal Cognitive Assessment (Raman et al., 2021). In a study of 35 COVID-19 patients with neurological manifestations five months after the acute phase, 29 % of the patients (n = 10) had neurocognitive test results corresponding to a severe impairment, whereas 54 % of the patients (n = 19) did not present any neurocognitive impairment. Abnormalities on MRI were found in 25 patients (71 %), white matter lesions being the most common finding. The MRI abnormalities were not associated, however, with the neurocognitive functioning (Hellgren et al., 2021). Interestingly, recent research has identified pathological volume changes in brain MRI, particularly in the basal ganglia region, associated with post-COVID-19 fatigue (Heine et al., 2023; Hafiz et al., 2022). However, these changes were not investigated in this study.

Despite the association between CMBs and the total cognitive score, CMBs in brain MRI may not be an independent predictor of lower total cognitive scores. Instead, the CMB burden in deep regions may serve as an indicator of the severity of acute COVID-19 disease, consistent with findings from previous studies (Dixon et al., 2020; Shoskes et al., 2022; McCarthy et al., 2023). A recent meta-analysis on brain MRI findings in COVID-19 patients reveals some associations between lower cognitive functioning and various brain MRI abnormalities, with the most common being white matter lesions and CMBs. However, no specific pattern in imaging findings has emerged (Boparai et al., 2023). Consistent with this, our data also indicate that severe WMHs (Fazekas scale 3) were associated with lower total cognitive scores, despite our limited sample size. The impact of COVID-19 on this association appears marginal and has been recognized in prior studies (Diez-Cirarda et al., 2023; Cecchetti et al., 2022; Boparai et al., 2023; Berdalin et al., 2020; Gunning-Dixon and Raz, 2000; Agarwal et al., 2020).

Hypertension, hypercholesterolemia, and diabetes are established risk factors for both CMBs and cognitive impairment (Newcombe et al., 2021; Debette and Markus, 2010), and also for the more severe COVID-19 ^38^, but we did not find an independent association between these underlying conditions and cognitive functioning. This finding is inconsistent with previous studies (Gorelick et al., 2011; Luchsinger and Mayeux, 2004; O'Brien et al., 2003) and may be explained by the limited sample size in our study, with few patients having multiple CMBs. Most likely, the complex interrelationship between these comorbidities, general patient demographics, risk of CMBs and COVID-19 severity explains the missing association. We also excluded patients with previous diagnoses of memory disorders or other major neurological diseases to be able to focus on COVID-19-related impairment.

This study has limitations. First, because no baseline data on cognitive functioning or imaging findings before COVID-19 were available, we cannot rule out the possibility that imaging findings or cognitive dysfunction existed before COVID-19. Our study did not include data on neurological symptoms or imaging findings in the acute phase of the disease either. Second, although we did not compare differences between groups, the patient groups differed regarding age and comorbidities, reflecting the risk factors for severe COVID-19; and regarding years of education. Additionally, the control group was not formally matched to the patient group. Third, as a single-center study, our study population was relatively small, and we cannot exclude a type 2 error. Fourth, we cannot exclude a volunteer bias. Fifth, a selection bias was possible as those with more extensive functional disability, and perhaps more findings in brain MRI, may have been less keen to participate, as well as the control group may have had a hidden motivation to undergo imaging. Additionally, the applicability of our findings may be limited to the first-wave variants of COVID-19 and the unvaccinated population. Strengths of this study are the prospective design, evaluation of cognitive functioning by a comprehensive neuropsychological assessment and MRI performed on patients from three different disease severity groups and non-COVID-19 controls.

In conclusion, we examined the association of cognitive functioning with brain MRI findings in both COVID-19 survivors and non-COVID-19 controls. Our primary observation was an association between the presence of CMBs and a lower total cognitive score, regardless of COVID-19 history. Among COVID-19 patients, those with 1–3 CMBs displayed lower total cognitive scores. Furthermore, a high burden of WMHs was associated with lower total cognitive scores, consistent with prior research. Our study provides additional insights into the unclear relationship between COVID-19-related imaging findings and cognitive functioning. We do not consider CMBs as self-explanatory factors in cognitive functioning, emphasizing the need for further research to clarify their actual impact in the context of COVID-19. Our results also suggest that long-lasting cognitive problems after COVID-19 probably have a complex and multifactorial etiology and do not necessarily have any unequivocal visible representation in MR imaging.

## CRediT authorship contribution statement

**Janne Pihlajamaa:** Writing – original draft. **Henriikka Ollila:** Writing – original draft, Investigation. **Juha Martola:** Writing – review & editing, Supervision, Investigation, Conceptualization. **Linda Kuusela:** Writing – review & editing, Investigation. **Riikka Pihlaja:** Writing – review & editing, Investigation. **Annamari Tuulio-Henriksson:** Writing – review & editing, Conceptualization. **Sanna Koskinen:** Writing – review & editing, Conceptualization. **Viljami Salmela:** Writing – review & editing, Formal analysis. **Laura Hokkanen:** Writing – review & editing, Conceptualization. **Marjaana Tiainen:** Writing – review & editing, Supervision, Investigation, Conceptualization. **Johanna Hästbacka:** Writing – review & editing, Supervision, Project administration, Investigation, Funding acquisition, Conceptualization.

## Funding

J.P. has received funding from Helsinki University Hospital Grant (Y780024023), J.H. has received Government Funding for University-level Research (TYH2021310) and 10.13039/501100004785NordForsk. H.O. has received funding from the 10.13039/501100007417Paulo Foundation and the 10.13039/100008723Finnish Medical Foundation (grant number 6417). The funders had no role in study design, data collection and analysis, decision to publish, or preparation of the manuscript. Open access funded by Helsinki University Library.

## Declaration of competing interest

The authors declare no competing interests.

## Data Availability

The data that has been used is confidential.
