# Supplementary material for: Cognitive functioning and brain MRI findings six months after acute COVID-19. A prospective observational study
Source: Neuroimage Rep. 2025 Mar 22;5(2):100254. doi: 10.1016/j.ynirp.2025.100254 (PMC12172750; doi:10.1016/j.ynirp.2025.100254)
Supplement: Multimedia component 1 [file mmc1.docx]

**Supplementary material
The association of the cognitive performance and brain MRI findings six months after acute COVID-19**

Janne Pihlajamaa, Henriikka Ollila, Juha Martola, Linda Kuusela, Riikka Pihlaja, Annamari S. Tuulio-Henriksson, Sanna K. Koskinen, Viljami Salmela, Laura Hokkanen, Marjaana Tiainen, Johanna Hästbacka,

**Table of contents:** 1

S1 Factors tested for association with cognition divided into different models 1

S2 Comparison of total cognition and CMB locations, COVID-19 patients only 2

S3 Comparison of memory and CMB locations, COVID-19 patients only 2
S4 Comparison of executive functions and CMB locations, COVID-19 patients only 3
S5 Comparison of attention and CMB locations, COVID-19 patients only 3
S6 Comparison of memory and CMB locations, ICU patients only 4
S7 Comparison of executive functions and CMB locations, ICU patients only 4
S8 Comparison of attention and CMB locations, ICU patients only 4

S9 ICU-related parameters tested for association with cognition 5

S10 Patient characteristics according to level of care 5

S11 Neuropsychological evaluation 6

S12 MRI Acquisition Parameters 7

S13 STROBE Statement—checklist of items that should be included in reports of 7 observational studies

| Table S1: Factors tested for association with cognition divided into different models | |  |  | |  | |  |
| --- | --- | --- | --- | --- | --- | --- | --- |
| **Model 1** | | R² = 0.466 |  | |  | |  |
| Predictor | | beta | SE | | t | | p |
| Age | | -0.438 | 0.0249 | | -8.18 | | < .001 |
| Education | | 0.453 | 0.1186 | | 8.53 | | < .001 |
| Male gender: | | -0.125 | 0.6193 | | -1.17 | | 0.244 |
|  | |  |  | |  | |  |
| **Model 2** | | R² = 0.484 |  | |  | |  |
| Predictor | | beta | SE | | t | | p |
| Age | | -0.3837 | 0.0268 | | -6.678 | | < .001 |
| Education | | 0.4366 | 0.1186 | | 8.225 | | < .001 |
| Male gender: | | -0.0899 | 0.6205 | | -0.837 | | 0.404 |
| Hypertension: | | -0.1728 | 0.7978 | | -1.251 | | 0.212 |
| Hypercholesterolemia: | | -0.1439 | 0.8910 | | -0.933 | | 0.352 |
| Diabetes: | | -0.1310 | 1.1487 | | -0.659 | | 0.511 |
|  | |  |  | |  | |  |
| **Model 3** | | R² = 0.484 |  | |  | |  |
| Predictor | | beta | SE | | t | | p |
| Age | | -0.3863 | 0.0272 | | -6.625 | | < .001 |
| Education | | 0.4358 | 0.1190 | | 8.179 | | < .001 |
| Male gender: | | -0.0918 | 0.6231 | | -0.851 | | 0.396 |
| Hypertension: | | -0.1688 | 0.8037 | | -1.213 | | 0.226 |
| Hypercholesterolemia: | | -0.1430 | 0.8933 | | -0.925 | | 0.356 |
| Diabetes: | | -0.1246 | 1.1589 | | -0.621 | | 0.535 |
| History of COVID-19: | | -0.0357 | 0.7218 | | -0.286 | | 0.775 |
|  | |  |  | |  | |  |
| **Model 4** | | R² = 0.514 |  | |  | |  |
| Predictor | | beta | SE | | t | | p |
| Age: | | -0.3552 | 0.0286 | | -5.779 | | < .001 |
| Education: | | 0.4110 | 0.1200 | | 7.649 | | < .001 |
| Male gender: | | -0.0957 | 0.6172 | | -0.896 | | 0.371 |
| Hypertension: | | -0.1850 | 0.8002 | | -1.335 | | 0.183 |
| Hypercholesterolemia: | | -0.0543 | 0.9082 | | -0.346 | | 0.730 |
| Diabetes: | | -0.1258 | 1.1715 | | -0.620 | | 0.536 |
| History of COVID-19: | | -0.0479 | 0.7199 | | -0.385 | | 0.701 |
| Fazekas: | |  |  | |  | |  |
| 1 – 0 | | -0.1316 | 0.8480 | | -0.896 | | 0.371 |
| 2 – 0 | | -0.2620 | 1.5978 | | -0.947 | | 0.345 |
| 3 – 0 | | -1.3465 | 2.6799 | | -2.903 | | 0.004 |
| CMB number: | |  |  | |  | |  |
| 1-3 | | -0.1858 | 0.7546 | | -1.422 | | 0.157 |
| ≥4 | | 0.0780 | 2.3887 | | 0.326 | | 0.745 |
|  |  | |  |  | |  | |
|  | |  |  | |  | |  |
| **Table S2: Comparison of total cognition and CMB locations, COVID-19 patients only (n=152)** | | R² = 0.479 |  | |  | |  |
| Predictor | | beta | SE | | t | | p |
| Age | | -0.37376 | 0.0332 | | -5.3338 | | < .001 |
| Education | | 0.46964 | 0.1486 | | 7.2966 | | < .001 |
| Male Gender: | | 0.00203 | 0.7708 | | 0.0154 | | 0.988 |
| Hypertension: | | -0.15804 | 0.9577 | | -0.9669 | | 0.335 |
| Hypercholesterolemia: | | -0.09680 | 1.0751 | | -0.5276 | | 0.599 |
| Diabetes: | | -0.21537 | 1.3327 | | -0.9469 | | 0.345 |
| Deep CMB: | | -0.18099 | 1.5019 | | -0.7061 | | 0.481 |
| Splenium CMB: | | 0.69012 | 2.2519 | | 1.7957 | | 0.075 |
| Lobar CMB: | | -0.35537 | 1.0056 | | -2.0707 | | 0.040 |
| Infratentorial CMB: | | 0.25476 | 1.5579 | | 0.9582 | | 0.340 |

† non-significant after FDR-correction

|  |  |  |  |  |
| --- | --- | --- | --- | --- |
| **Table S3: Comparison of memory and CMB locations, COVID-19 patients only** | R² = 0.389 |  |  |  |
| Predictor | beta | SE | t | p |
| Age | -0.3736 | 0.0150 | -4.921 | < .001 |
| Education | 0.3868 | 0.0670 | 5.547 | < .001 |
| Male Gender: | -0.0344 | 0.3475 | -0.241 | 0.810 |
| Hypertension: | -0.1631 | 0.4317 | -0.921 | 0.359 |
| Hypercholesterolemia: | 0.0793 | 0.4846 | 0.399 | 0.691 |
| Diabetes: | -0.3441 | 0.6008 | -1.396 | 0.165 |
| Deep CMB: | -0.0876 | 0.6770 | -0.316 | 0.753 |
| Splenium CMB: | 0.4823 | 1.0151 | 1.158 | 0.249 |
| Lobar CMB: | -0.2159 | 0.4533 | -1.161 | 0.248 |
| Infratentorial CMB: | 0.1192 | 0.7023 | 0.414 | 0.680 |

|  |  |  |  |  |
| --- | --- | --- | --- | --- |
| **Table S4: Comparison of executive functions and CMB locations, COVID-19 patients only** | R² = 0.405 |  |  |  |
| Predictor | beta | SE | t | p |
| Age | 0.0133 | -4.443 | -4.208 | < .001 |
| Education | 0.0596 | 6.407 | 6.145 | < .001 |
| Male Gender: | 0.3094 | 0.141 | -0.262 | 0.888 |
| Hypertension: | 0.3844 | -0.794 | -0.934 | 0.428 |
| Hypercholesterolemia: | 0.4316 | -0.749 | -0.709 | 0.455 |
| Diabetes: | 0.5350 | -0.626 | -0.841 | 0.532 |
| Deep CMB: | 0.6029 | -0.149 | -0.114 | 0.882 |
| Splenium CMB: | 0.9040 | 1.728 | 1.736 | 0.086 |
| Lobar CMB: | 0.4037 | -2.003 | -1.974 | 0.047† |
| Infratentorial CMB: | 0.6254 | 0.720 | 0.824 | 0.473 |

† non-significant after FDR-correction

|  |  |  |  |  |
| --- | --- | --- | --- | --- |
| **Table S5: Comparison of attention and CMB locations, COVID-19 patients only** | R² = 0.318 |  |  |  |
| Predictor | beta | SE | t | p |
| Age | -0.2627 | 0.0135 | -2.980 | 0.001 |
| Education | 0.4025 | 0.0605 | 5.325 | < .001 |
| Male Gender: | 0.0252 | 0.3138 | -0.181 | 0.868 |
| Hypertension: | -0.1082 | 0.3899 | -0.550 | 0.564 |
| Hypercholesterolemia: | -0.2091 | 0.4377 | -0.875 | 0.321 |
| Diabetes: | -0.0422 | 0.5426 | -0.420 | 0.872 |
| Deep CMB: | -0.3616 | 0.6115 | -1.166 | 0.220 |
| Splenium CMB: | 0.6247 | 0.9169 | 1.384 | 0.158 |
| Lobar CMB: | -0.3587 | 0.4094 | -1.579 | 0.070 |
| Infratentorial CMB: | 0.3622 | 0.6343 | 0.824 | 0.236 |

|  |  |  |  |  |
| --- | --- | --- | --- | --- |
| **Table S6: Comparison of memory and CMB locations, ICU patients only** | R² = 0.360 |  |  |  |
| Predictor | beta | SE | t | p |
| Age | -0.319 | 0.0245 | -2.700 | 0.009 |
| Education | 0.429 | 0.0970 | 3.641 | < .001 |
| Male Gender: | 0.250 | 0.5682 | 0.959 | 0.342 |
| Hypertension: | -0.132 | 0.5756 | -0.499 | 0.620 |
| Hypercholesterolemia: | 0.156 | 0.6854 | 0.494 | 0.623 |
| Diabetes: | -0.353 | 0.8206 | -0.937 | 0.353 |
| Deep CMB: | -0.280 | 0.9389 | -0.649 | 0.519 |
| Splenium CMB: | 0.755 | 1.2425 | 1.324 | 0.191 |
| Lobar CMB: | -0.282 | 0.6616 | -0.930 | 0.357 |
| Infratentorial CMB: | 0.530 | 1.4002 | 0.824 | 0.413 |

|  |  |  |  |  |
| --- | --- | --- | --- | --- |
| **Table S7: Comparison of executive functions and CMB locations, ICU patients only** | R² = 0.487 |  |  |  |
| Predictor | beta | SE | t | p |
| Age | -0.2687 | 0.0268 | -2.544 | 0.014 |
| Education | 0.3517 | 0.1063 | 3.334 | 0.002 |
| Male Gender: | -0.2282 | 0.6224 | -0.976 | 0.333 |
| Hypertension: | -0.0824 | 0.6306 | -0.348 | 0.729 |
| Hypercholesterolemia: | 0.3317 | 0.7508 | 1.176 | 0.245 |
| Diabetes: | -0.8063 | 0.8990 | -2.389 | 0.021 |
| Deep CMB: | 0.1624 | 1.0285 | 0.420 | 0.676 |
| Splenium CMB: | 1.0334 | 1.3611 | 2.022 | 0.048† |
| Lobar CMB: | -0.7449 | 0.7248 | -2.737 | 0.008 |
| Infratentorial CMB: | -0.0863 | 1.5338 | -0.150 | 0.881 |

† non-significant after FDR-correction

|  |  |  |  |  |
| --- | --- | --- | --- | --- |
| **Table S8: Comparison of attention and CMB locations, ICU patients only** | R² = 0.310 |  |  |  |
| Predictor | beta | SE | t | p |
| Age | -0.1881 | 0.0263 | -1.5347 | 0.131 |
| Education | 0.2241 | 0.1041 | 1.8320 | 0.073 |
| Male Gender: | 0.0139 | 0.6097 | 0.0512 | 0.959 |
| Hypertension: | -0.1616 | 0.6178 | -0.5885 | 0.559 |
| Hypercholesterolemia: | -0.0568 | 0.7356 | -0.1736 | 0.863 |
| Diabetes: | -0.6535 | 0.8807 | -1.6688 | 0.101 |
| Deep CMB: | -0.2743 | 1.0076 | -0.6124 | 0.543 |
| Splenium CMB: | 0.8510 | 1.3334 | 1.4355 | 0.157 |
| Lobar CMB: | -0.5161 | 0.7100 | -1.6349 | 0.108 |
| Infratentorial CMB: | 0.2558 | 1.5026 | 0.3829 | 0.703 |

|  |  |  |  |  |
| --- | --- | --- | --- | --- |
| **Table S9: ICU-related parameters tested for association with cognition** | R² = 0.437 |  |  |  |
| Predictor | beta | SE | t | p |
| Age | -0.2086 | 0.0648 | -3.2172 | 0.002 |
| Education | 0.8275 | 0.2539 | 3.2595 | 0.002 |
| Male Gender: | -0.5821 | 1.5033 | -0.3872 | 0.700 |
| Hypertension: | -1.7289 | 1.5067 | -1.1475 | 0.256 |
| Hypercholesterolemia: | -1.0269 | 1.6244 | -0.6322 | 0.530 |
| Diabetes: | -1.9898 | 1.9234 | -1.0346 | 0.305 |
| ICU length of stay: | 0.0365 | 0.0937 | 0.3895 | 0.698 |
| Mechanical ventilation: | -0.0157 | 1.7827 | -0.0088 | 0.993 |
| Proning episodes: | -0.6692 | 0.5923 | -1.1298 | 0.263 |

**S10 Patient characteristics according to level of care**

| **Level of care:** | **ICU, n=67** | **WARD, n = 44** | **HOME, n = 44** | **CONTROL n = 48** | **χ²/F** | **p** |
| --- | --- | --- | --- | --- | --- | --- |
| **Sex male, n (%)** | 43 (64) | 18 (41) | 12 (27) | 25 (52) | 15.7 | 0.001 |
| **Age, years, median (IQR)** | 59 (44–74) | 57 (44–70) | 45 (27–63) | 56 (42–70) | 29.1 | < 0.001 |
| **Education years, median (IQR)** | 14 (11-17) | 15 (11-19) | 15 (12-18) | 15 (11-19) | 14.7 | 0.002 |
| **Hypertension, n (%)** | 37 (55) | 13 (30) | 8 (18) | 11 (23) | 21.3 | < 0.001 |
| **Hypercholesterolemia, n (%)** | 22 (33) | 10 (23) | 4 (9) | 7 (15) | 10.6 | 0.014 |
| **Diabetes, n (%)** | 16 (24) | 5 (11) | 2 (5) | 1 (2) | 15.9 | 0.001 |
|  |  |  |  |  |  |  |
| **CMB number** |  |  | ‡ |  | 9.27 | 0.026 |
| **0 CMB, n (%)** | 41 (61) | 31 (70) | 37 (84) | 38 (79) | 8.45 | 0.038 |
| **1-3 CMBs, n (%)** | 17 (25) | 9 (20) | 7 (16) | 10 (21) | 4.82 | 0.008 |
| **≥4 CMBs, n (%)** | 8 (12) | 4 (9) | 0 (0) | 0 (0) | 3.42 | 0.055 |
| **CMB location** |  |  |  |  |  |  |
| **Deep, n (%)** | 4 (6) | 2 (5) | 0 (0) | 1 (2) | 3.26 | 0.012 |
| **Splenial, n (%)** | 9 (13) | 0 (0) | 0 (0) | 0 (0) | 19.31 | 0.096 |
| **Lobar, n (%)** | 9 (13) | 6 (14) | 3 (7) | 7 (15) | 1.60 | 0.008 |
| **Infratentorial, n (%)** | 0 (0) | 1 (2) | 2 (5) | 1 (2) | 2.87 | 0.014 |
|  |  |  |  |  |  |  |
| **Fazekas scale, n (%)** |  |  | ‡ |  | 37.1 | **< 0.001** |
| **0, n (%)** | 7 (10) | 4 (9) | 14 (32) | 8 (17) |  |  |
| **1, n (%)** | 51 (7) | 38 (86) | 27 (61) | 38 (79) |  |  |
| **2, n (%)** | 6 (9) | 2 (5) | 3 (7) | 1 (2) |  |  |
| **3, n (%)** | 2 (3) | 0 (0) | 0 (0) | 1 (2) |  |  |
| **Infarctions, n (%):** | 4 (6) | 0 (0) | 0 (0) | 0 (0) | 8.24 | 0.041 |
| **Total cognitive score, median (IQR)** | -1.3 (-10.5– -7.9) | 0.2 (-8.1–8.5) | 4.7 (0.1–9.3) | 0.8 (-7.4–8.4) |  | 0.176 |
| **Memory, median (IQR)** | -1.1 (-4.4– -0.1) | 0.0 (-3.6–3.6) | 2.0 (-0.2–4.2) | 0.1 (-3.0–3.2) |  |  |
| **Attention, median (IQR)** | -0.7 (-4.5–2.8) | 0.5 (-2.4–3.4) | 1.1 (-0.8–3.0) | 0.5 (-2.0–3.0) |  |  |
| **Executive functioning, median (IQR)** | -0.3 (-3.2–3.6) | 0.1 (-3.2–3.4) | 1.5 (0.4–2.6) | 0.7 (-1.5–3.2) |  |  |

IQR interquartile range, ICU intensive care unit, CMB cerebral microbleed.

‡ One patient in this group had DAI and was therefore excluded from this category

S11 Neuropsychological evaluation

Cognitive Functioning

The primary outcome of this study was cognitive functioning assessed six months after hospital discharge, using a comprehensive neuropsychological evaluation. The control group underwent the same evaluation in the spring of 2021. We selected key outcome measures to assess three cognitive domains: memory, executive functions, and attention. Attention was measured using the Wechsler Adult Intelligence Scale-IV coding [1], the Continuous Performance Test [2,3], and Stroop Naming [4]. Executive functions were assessed with Trail Making Test B [5], Stroop Interference [6], and the Frontal Assessment Battery [6]. Memory was evaluated using the Wechsler Memory Scale version III (WMS-III) word list, delayed recall, WMS-III logical memory, delayed recall [7], and Rey Complex Figure delayed recall [8]. The results were standardized into Z-scores [9], and a total cognitive score was calculated by summing the domain scores. In all assessments, higher scores indicate better cognitive performance.

1. Wechsler D. WAIS-IV: Wechsler Adult Intelligence Scale [Finnish version] 4. Helsinki: Psykologien Kustannus Oy; 2012. [[Google Scholar](https://scholar.google.com/scholar_lookup?title=WAIS-IV:%20Wechsler%20Adult%20Intelligence%20Scale%20%5BFinnish%20version%5D&author=D%20Wechsler&publication_year=2012&)]
2. Mueller ST, Piper BJ. The psychology experiment building language (PEBL) and PEBL test battery. J Neurosci Methods. 2014;222:250–259. doi: 10.1016/j.jneumeth.2013.10.024. [[DOI](https://doi.org/10.1016/j.jneumeth.2013.10.024)] [[PMC free article](https://pmc.ncbi.nlm.nih.gov/articles/PMC3897935/)] [[PubMed](https://pubmed.ncbi.nlm.nih.gov/24269254/)] [[Google Scholar](https://scholar.google.com/scholar_lookup?journal=J%20Neurosci%20Methods&title=The%20psychology%20experiment%20building%20language%20(PEBL)%20and%20PEBL%20test%20battery&author=ST%20Mueller&author=BJ%20Piper&volume=222&publication_year=2014&pages=250-259&pmid=24269254&doi=10.1016/j.jneumeth.2013.10.024&)]
3. Conners CK, Epstein JN, Angold A, Klaric J. Continuous performance test performance in a normative epidemiological sample. J Abnorm Child Psychol. 2003;31(5):555–562. doi: 10.1023/A:1025457300409. [[DOI](https://doi.org/10.1023/A:1025457300409)] [[PubMed](https://pubmed.ncbi.nlm.nih.gov/14561062/)] [[Google Scholar](https://scholar.google.com/scholar_lookup?journal=J%20Abnorm%20Child%20Psychol&title=Continuous%20performance%20test%20performance%20in%20a%20normative%20epidemiological%20sample&author=CK%20Conners&author=JN%20Epstein&author=A%20Angold&author=J%20Klaric&volume=31&issue=5&publication_year=2003&pages=555-562&pmid=14561062&doi=10.1023/A:1025457300409&)]
4. Stroop JR. Studies of interference in serial verbal reactions. J Exp Psychol. 1935;18(6):643–662. doi: 10.1037/h0054651. [[DOI](https://doi.org/10.1037/h0054651)] [[Google Scholar](https://scholar.google.com/scholar_lookup?journal=J%20Exp%20Psychol&title=Studies%20of%20interference%20in%20serial%20verbal%20reactions&author=JR%20Stroop&volume=18&issue=6&publication_year=1935&pages=643-662&doi=10.1037/h0054651&)]
5. Reitan RM. The relation of the trail making test to organic brain damage. J Consult Psychol. 1955;19(5):393–394. doi: 10.1037/h0044509. [[DOI](https://doi.org/10.1037/h0044509)] [[PubMed](https://pubmed.ncbi.nlm.nih.gov/13263471/)] [[Google Scholar](https://scholar.google.com/scholar_lookup?journal=J%20Consult%20Psychol&title=The%20relation%20of%20the%20trail%20making%20test%20to%20organic%20brain%20damage&author=RM%20Reitan&volume=19&issue=5&publication_year=1955&pages=393-394&pmid=13263471&doi=10.1037/h0044509&)]
6. Dubois B, Slachevsky A, Litvan I, Pillon B. The FAB: a frontal assessment battery at bedside. Neurology. 2000;55(11):1621–1626. doi: 10.1212/WNL.55.11.1621. [[DOI](https://doi.org/10.1212/WNL.55.11.1621)] [[PubMed](https://pubmed.ncbi.nlm.nih.gov/11113214/)] [[Google Scholar](https://scholar.google.com/scholar_lookup?journal=Neurology&title=The%20FAB:%20a%20frontal%20assessment%20battery%20at%20bedside&author=B%20Dubois&author=A%20Slachevsky&author=I%20Litvan&author=B%20Pillon&volume=55&issue=11&publication_year=2000&pages=1621-1626&pmid=11113214&doi=10.1212/WNL.55.11.1621&)]
7. Wechsler D. WMS-III: Wechsler Memory Scale [Finnish version] 3. Helsinki: Psykologien Kustannus Oy; 2008. [[Google Scholar](https://scholar.google.com/scholar_lookup?title=WMS-III:%20Wechsler%20Memory%20Scale%20%5BFinnish%20version%5D&author=D%20Wechsler&publication_year=2008&)]
8. Corwin J, Bylsma FW. Psychological examination of traumatic encephalopathy. Clin Neuropsychol. 1993;7(1):3–21. doi: 10.1080/13854049308401883. [[DOI](https://doi.org/10.1080/13854049308401883)] [[Google Scholar](https://scholar.google.com/scholar_lookup?journal=Clin%20Neuropsychol&title=Psychological%20examination%20of%20traumatic%20encephalopathy&author=J%20Corwin&author=FW%20Bylsma&volume=7&issue=1&publication_year=1993&pages=3-21&doi=10.1080/13854049308401883&)]
9. Ollila, H., et al., Long-term cognitive functioning is impaired in ICU-treated COVID-19 patients: a comprehensive controlled neuropsychological study. Crit Care, 2022. 26(1): p. 223.

**S12 MRI Acquisition Parameters**

| **Sequence name** | **TR (ms)** | **TE (ms)** | **Flip angle** | **Resolution (mm)** | **Slice thickness (mm)** | **FOV (mm)** | **Slices** | **Other parameters** | **Duration (mm:ss)** |
| --- | --- | --- | --- | --- | --- | --- | --- | --- | --- |
| **T1 3D** | 2400 | 4,6 | 8 | 0.8x0.8 | 0,8 | 240x240 | 198 | SENSE= 2 (AP) | 6:16 |
| **T2 3D** | 2500 | 331 | 90 | 0.8x0.85 | 0,8 | 230x230 | 400 | TSE=117, SENSE=2(P) 1.5 (RL) | 5:07 |
| **3D FLAIR** | 4800 | 320 | 40 | 1.0x1.2 | 1,2 | 240x240 | 283 | TSE=140, SENSE=1.5(P) 2 (RL) | 4:48 |
| **SWI** | 31 | 7.2, 13.4, 19.6, 25.8 | 17 | 0.6x0.6 | 2 | 240x194 |  | CS=4 | 3:31 |
| **T2 TSE SPIR** | 4200 | 80 | 90 | 0.5x0.6 | 2 | 170x172 | 48 | TSE=15, SENSE=1.5(RL) | 3:38 |
| **T2 3D DRIVE** | 2000 | 120 | 90 | 0.4x0.55 | 1 | 130x130 | 100 | TSE=56, CS=4.5 | 4:00 |
| **COW MRA** | 23 | 3,5 | 18 | 0.4x0.6 | 1,2 | 230x180 | 220 | CS=4.5 | 5:06 |

**S13** **STROBE Statement—checklist of items that should be included in reports of observational studies**

|  | **Item No.** | **Recommendation** | **Page No.** |
| --- | --- | --- | --- |
| **Title and abstract** | **1** | (a) Indicate the study’s design with a commonly used term in the title or the abstract | **2** |
|  |  | (b) Provide in the abstract an informative and balanced summary of what was done and what was found | **2** |
| **Introduction** |  |  |  |
| **Background/rationale** | **2** | Explain the scientific background and rationale for the investigation being reported | **3** |
| **Objectives** | **3** | State specific objectives, including any prespecified hypotheses | **4-5** |
| **Methods** |  |  |  |
| **Study design** | **4** | Present key elements of study design early in the paper | **4** |
| **Setting** | **5** | Describe the setting, locations, and relevant dates, including periods of recruitment, exposure, follow-up, and data collection | **4-5** |
| **Participants** | **6** | (a) Cohort study—Give the eligibility criteria, and the sources and methods of selection of participants. Describe methods of follow-up | **5-6** |
|  |  | Case-control study—Give the eligibility criteria, and the sources and methods of case ascertainment and control selection. Give the rationale for the choice of cases and controls |  |
|  |  | Cross-sectional study—Give the eligibility criteria, and the sources and methods of selection of participants |  |
|  |  | (b) Cohort study—For matched studies, give matching criteria and number of exposed and unexposed |  |
|  |  | Case-control study—For matched studies, give matching criteria and the number of controls per case |  |
| **Variables** | **7** | Clearly define all outcomes, exposures, predictors, potential confounders, and effect modifiers. Give diagnostic criteria, if applicable | **5-7** |
| **Data sources/ measurement** | **8*** | For each variable of interest, give sources of data and details of methods of assessment (measurement). Describe comparability of assessment methods if there is more than one group | **5-7** |
| **Bias** | **9** | Describe any efforts to address potential sources of bias | **5-7** |
| **Study size** | **10** | Explain how the study size was arrived at | **5-7** |
| **Quantitative variables** | **11** | Explain how quantitative variables were handled in the analyses. If applicable, describe which groupings were chosen and why | **5-7** |
| **Statistical methods** | **12** | (a) Describe all statistical methods, including those used to control for confounding | **6-7** |
|  |  | (b) Describe any methods used to examine subgroups and interactions | **6-7** |
|  |  | (c) Explain how missing data were addressed | **6-7** |
|  |  | (d) Cohort study—If applicable, explain how loss to follow-up was addressed | **6-7** |
|  |  | Case-control study—If applicable, explain how matching of cases and controls was addressed |  |
|  |  | Cross-sectional study—If applicable, describe analytical methods taking account of sampling strategy |  |
|  |  | (e) Describe any sensitivity analyses |  |
| **Results** |  |  |  |
| **Participants** | **13*** | (a) Report numbers of individuals at each stage of study—eg numbers potentially eligible, examined for eligibility, confirmed eligible, included in the study, completing follow-up, and analysed | **7-10** |
|  |  | (b) Give reasons for non-participation at each stage | **7-12** |
|  |  | (c) Consider use of a flow diagram | **9** |
| **Descriptive data** | **14*** | (a) Give characteristics of study participants (eg demographic, clinical, social) and information on exposures and potential confounders | **7-10** |
|  |  | (b) Indicate number of participants with missing data for each variable of interest | **7-10** |
|  |  | (c) Cohort study—Summarise follow-up time (eg, average and total amount) |  |
| **Outcome data** | **15*** | Cohort study—Report numbers of outcome events or summary measures over time |  |
|  |  | Case-control study—Report numbers in each exposure category, or summary measures of exposure |  |
|  |  | Cross-sectional study—Report numbers of outcome events or summary measures | **7-12** |
| **Main results** | **16** | (a) Give unadjusted estimates and, if applicable, confounder-adjusted estimates and their precision (eg, 95% confidence interval). Make clear which confounders were adjusted for and why they were included | **7-12** |
|  |  | (b) Report category boundaries when continuous variables were categorized | **7-12** |
|  |  | (c) If relevant, consider translating estimates of relative risk into absolute risk for a meaningful time period | **7-12** |
| **Other analyses** | **17** | Report other analyses done—eg analyses of subgroups and interactions, and sensitivity analyses | **7-12 + supplementary material** |
| **Discussion** |  |  |  |
| **Key results** | **18** | Summarise key results with reference to study objectives | **13** |
| **Limitations** | **19** | Discuss limitations of the study, taking into account sources of potential bias or imprecision. Discuss both direction and magnitude of any potential bias | **14-15** |
| **Interpretation** | **20** | Give a cautious overall interpretation of results considering objectives, limitations, multiplicity of analyses, results from similar studies, and other relevant evidence | **15** |
| **Generalisability** | **21** | Discuss the generalisability (external validity) of the study results | **15** |
| **Other information** |  |  |  |
| **Funding** | **22** | Give the source of funding and the role of the funders for the present study and, if applicable, for the original study on which the present article is based | **15** |
